# A Rare Case of Drug-Induced Liver Injury (DILI) From Topiramate

**DOI:** 10.7759/cureus.81120

**Published:** 2025-03-24

**Authors:** Raymart Macasaet, FNU Payal, Karan Yagnik, Malay Rathod, David Aquino, Shilpan Shah

**Affiliations:** 1 Internal Medicine, Monmouth Medical Center, Long Branch, USA; 2 Medicine, Rutgers Monmouth Hospital, New Jersey, USA

**Keywords:** cholestatic liver injury, drug-induced liver injury, hepatotoxic drugs, phentermine-topiramate, transaminitis-raised liver enzyme

## Abstract

A 72-year-old male individual with a past medical history of seizures and ongoing management with topiramate for two months presented at the emergency department with jaundice. Ten days before the presentation, the patient started feeling fatigued and had intermittent generalized mild pain (grade 3/10) with dark-brown discoloration of urine. On physical examination, he was normotensive at 124/54 with a pulse rate of 72 beats per minute. His weight was 73.9 kg, with an average body mass index of 20.93 kg/m2. Physical examination revealed scleral icterus and diffuse jaundice all over his body, otherwise unremarkable. On laboratory work-up, the patient had elevations in alanine aminotransferase at 74.6 U/L and aspartate aminotransferase at 498 U/L. He also had extremely high alkaline phosphatase (ALP) at 1,353 U/L, total bilirubin was 10.9 mg/dL while his direct bilirubin was 8.1 mg/dL, lactate dehydrogenase (LDH) was mildly high at 300 U/L, Gamma-glutamyl transferase (GGT) was high at 1,274 U/L. Ammonia levels were found to be mildly high at 48 umol/L. His hemoglobin was 12.3 g/dL, at his baseline. Carbohydrate antigen 19-9 was slightly high at 71 U/mL, and alpha-fetoprotein was high at 10.9 ng/mL. An ultrasound of the abdomen showed no evidence of gallstone, gallbladder wall thickening, or common duct dilatation. Computed tomography (CT) scan with intravenous contrast of the abdomen and pelvis showed hepatic steatosis with borderline size liver (17 cm). A previous CT scan two months prior also showed normal liver and gallbladder. Magnetic resonance imaging (MRI) and magnetic resonance cholangiopancreatography (MRCP) showed consistent results with CT scans. Topiramate was then tapered and then discontinued. During follow-up three months after discharge, his alanine aminotransferase (ALT), aspartate aminotransferase (AST), ALP, total bilirubin, and direct bilirubin all came back to normal.

Drug-induced liver injury with topiramate is rare. Most previous studies and case reports have presented liver injury with topiramate only when combined with other antiepileptic drugs (AEDs), antipsychotics, or other hepatotoxic drugs. However, the case shows that topiramate can independently cause drug-induced liver injury. The timing of the onset of jaundice in our patient, coinciding with the initiation of topiramate, suggests a possible drug-induced liver injury, and so does discontinuation of the drug improved liver enzymes. Given that only a tiny percentage of patients on topiramate develop significant liver injury, this case highlights the need for vigilance in monitoring liver function in patients initiating this medication.

## Introduction

Topiramate is an antiepileptic medication that exerts its effects by blocking voltage-dependent sodium channels, enhancing gamma-aminobutyric acid A (GABA-A) receptor activity, and inhibiting glutamate activity [1]. It is approved for use as both a monotherapy and adjunctive therapy in the treatment of primary generalized tonic-clonic and partial-onset seizures, as well as for migraine prevention in adults. Off-label uses include the management of neuropathic pain, weight loss, and alcohol and tobacco dependence [2]. Adverse events associated with topiramate are dose-dependent and include acute myopia, metabolic acidosis, suicidal behavior, hyperthermia, oligohidrosis, and congenital malformations. Overdose or toxicity symptoms may manifest as sedation, dysarthria, blurred vision, gait disturbances, abdominal pain, and agitation [3].

While topiramate is rarely associated with liver injury, the risk increases when it is used concomitantly with other antiepileptic, psychiatric, or hepatotoxic medications. The mechanism of liver injury involves the induction of CYP3A4 and inhibition of CYP2C19 enzymes. Idiosyncratic drug-induced liver injury (DILI) is defined as an unintended, unpredictable response to a drug occurring at recommended therapeutic doses. Common causes of DILI include acetaminophen, antibacterials (such as penicillins and cephalosporins), isoniazid, ketoconazole, and nonsteroidal anti-inflammatory drugs (NSAIDs) [4]. The incidence of topiramate-induced liver injury is estimated to be around 1% in patients taking the drug. From September 2003 to May 2013, only two cases of topiramate-associated DILI were reported among 899 confirmed DILI cases in the Drug-Induced Liver Injury Network (DILIN) prospective study [5].

## Case presentation

A 72-year-old male individual with a past medical history of paroxysmal atrial fibrillation, hypertension, seizures with ongoing management with topiramate for two months already, and surgical history of right inguinal mesh explantation and debridement presented at the emergency department (ED) with jaundice. Ten days before the consult, the patient started feeling fatigued and had intermittent generalized mild pain (grade 3/10) with dark-brown discoloration of urine. The patient denied nausea, vomiting, diarrhea, constipation, fever, chills, rectal pain, anal bleeding, blood in stool, and abdominal distension. The patient denied recent travel, exposure to someone sick, and eating raw or unusual food. The patient claimed he has been drinking green tea but has used it for over 20 years. On physical examination, he was normotensive at 124/54 with a pulse rate of 72. He was afebrile at 97.2°F, saturating at 97% on room air and breathing 20 cycles per minute. His weight was 73.9 kg, with an average BMI of 20.93 kg/m2. He had scleral icterus and diffuse jaundice all over his body. The abdomen was flat and soft with normal bowel sounds. No hernia nor other masses palpated. There was no hepatosplenomegaly, spider angiomas, telangiectasias, and caput medusae. Murphy’s sign was negative.

On laboratory work-up, the patient had elevations in alanine aminotransferase (ALT) at 74.6 U/L (previously mildly elevated at 45 U/L two months ago) and aspartate aminotransferase at 498 U/L (previously normal at 26 U/L two months ago). He also had extremely high alkaline phosphatase (ALP) at 1,353 U/L (previously normal at 53 U/L two months prior). His total bilirubin was 10.9 mg/dL, elevated from 0.6 taken two months prior, while his direct bilirubin was 8.1 mg/dL with baseline unknown. His lactate dehydrogenase (LDH) was mildly high at 300 U/L, while his lactate was low at 0.4 mmol/L. Gamma-glutamyl transferase (GGT) was high at 1,274 U/L. Ammonia levels were found to be mildly high at 48 umol/L. He also had erythrocytopenia at 3.98 10^6/uL and low hemoglobin at 12.3 g/dL, which were both chronic. His coagulation profile was also deranged but had been chronic. Both prothrombin time (PT) and activated partial thromboplastin time (aPTT) were high at 14.2 seconds and 42.8 seconds, respectively. The International Normalized Ratio (INR) was high at 1.26. Acetaminophen level was low at 4 ug/mL, and salicylate was also low at <3 mg/dL. His serum alcohol was normal, and he had negative urine toxicology screening. Urinalysis showed dark-yellow urine with moderate bilirubin, positive ictotest, amorphous, and calcium oxalate crystals.

Carcinoembryonic antigen, ceruloplasmin, actin antibody, antinuclear antibody, and mitochondrial antibody were negative. Hepatitis panel, cytomegalovirus, human immunodeficiency virus, herpes simplex virus 1 and 2, and Epstein-Barr virus were also negative. Carbohydrate antigen 19-9 was slightly high at 71 U/mL, and alpha-fetoprotein was high at 10.9 ng/mL.

**Figure 1:**
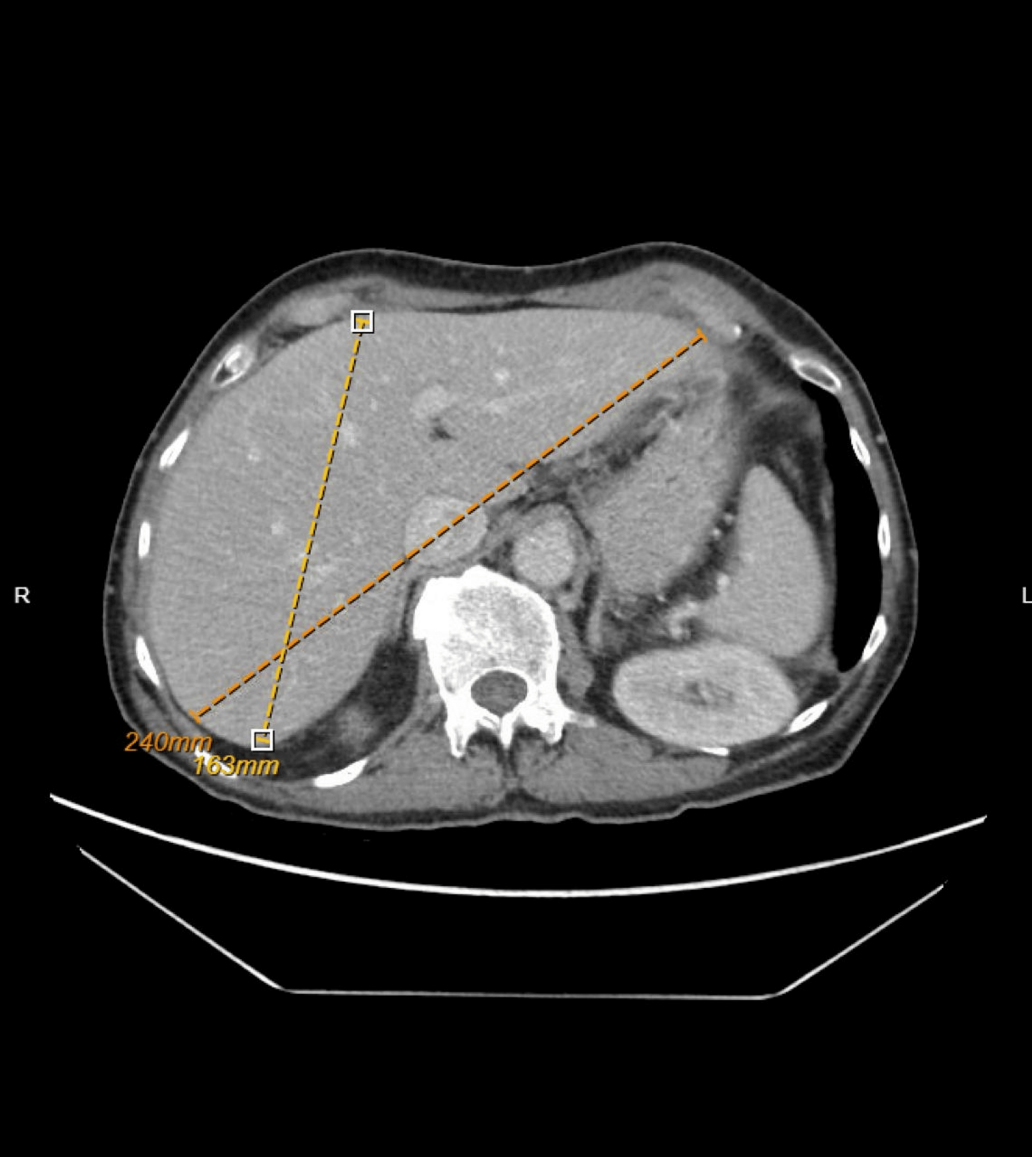
CTAP showing hepatic steatosis. CTAP, computed tomography arterial portography.

**Figure 2:**
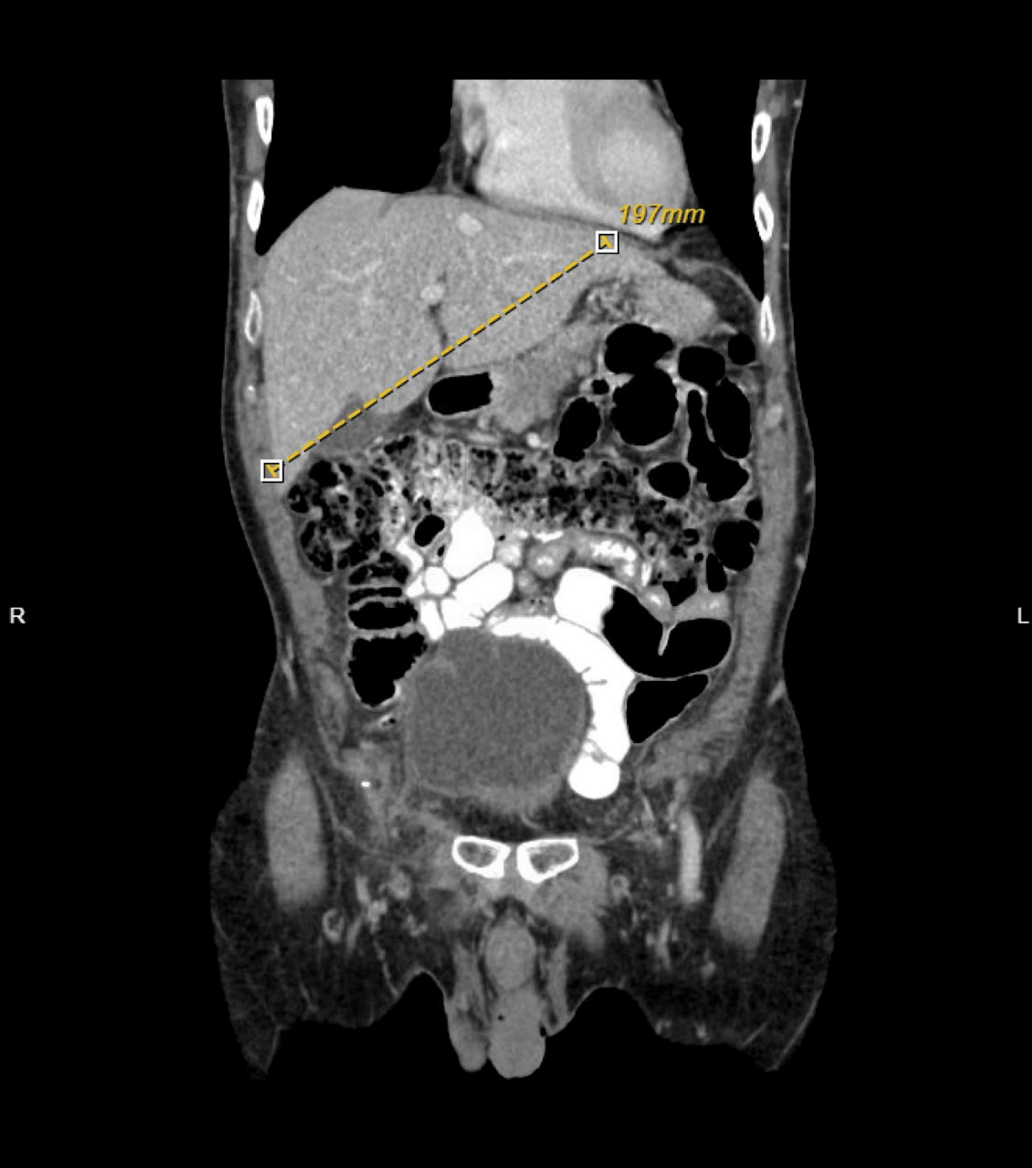
CTAP showing hepatic enlargement. CTAP, computed tomography arterial portography.

An ultrasound of the abdomen was done, which showed no evidence of gallstone, gallbladder wall thickening, or common duct dilatation. A computed tomography (CT) scan with intravenous contrast of the abdomen and pelvis was done, which showed hepatic steatosis with borderline-size liver (17 cm) (Figures 1, 2). There was no evidence of radiopaque cholelithiasis or ductal dilatation. A previous CT scan two months prior also showed normal liver and gallbladder. Magnetic resonance imaging (MRI) and magnetic resonance cholangiopancreatography (MRCP) showed consistent results with CT scans. There was no cholelithiasis, choledocholithiasis, or biliary ductal dilation. The pancreas was normal, and no pancreatic mass or pancreatic ductal dilation was seen. However, MRI/MRCP noted negative steatosis in the liver. The liver did not show any masses. Endoscopy and endoscopic retrograde cholangiopancreatography (ERCP) were not done as there are no signs of intra or extrahepatic biliary obstruction that would benefit from or warrant endoscopic ultrasound (EUS)/ERCP.

Topiramate was then tapered from 50 mg twice daily to 25 mg twice daily for one week, then 25 mg daily for a week, and then discontinued. During follow-up, three months after discharge, his ALT, AST, ALP, total bilirubin, and direct bilirubin all came back to normal. The patient had a follow-up with a neurologist for seizures, and he is currently not on any anti-seizure medications.

## Discussion

Topiramate is a newer antiepileptic drug (AED) that is effective for both partial and generalized tonic-clonic seizures, migraine headaches, and bipolar disorder. Herein, we report a case of a patient who developed painless jaundice and elevated liver function tests (LFTs) following the initiation of topiramate for tonic-clonic seizures. This patient was admitted for a hernia repair in February 2024 and found to have elevated LFTs despite being asymptomatic. Before starting topiramate in December 2023, the patient's LFTs were normal.

Upon admission, the patient exhibited significantly elevated LFTs, with ALT (746 U/L) markedly higher than AST (498 U/L). The elevation in liver enzymes was unexpected, given the absence of typical etiologies such as alcohol use or a history of hepatitis. Workup, including but not limited to viral hepatitis, autoimmune panels, and imaging studies, including MRCP, MRI, and CT abdomen, could not identify any pathology or obstruction. Additionally, after discontinuation of the topiramate, the patient's LFTs improved.

Previously available literature has been documented as topiramate to be a culprit of reversible liver injury in sporadic instances. Reports indicate that the rate of DILI with topiramate is rare, although most cases are mild [6]. Most previous studies and case reports have presented liver injury with topiramate only when combined with other AEDs, antipsychotics, or other hepatotoxic drugs [7-9]. That is mainly because of the induction of CYP3A4 or inhibiting CYP2C19 enzymes [9].

Topiramate was associated with liver injury in approximately 1.3% of patients, and the injury often manifested after a few months of treatment [10]. This suggests that topiramate alone may also cause DILI. In animal studies, Huang et al. (2007) observed that it can decrease glutathione levels, hence, decreasing the antioxidant capacities of the organism [11]. This explains idiosyncratic DILI with topiramate. However, Schmidt and Siemes (1998) do not recommend routine monitoring of LFTs with topiramate therapy [12].

The timing of the onset of jaundice in our patient, coinciding with the initiation of topiramate, suggests a possible drug-induced liver injury, and so does discontinuation of the drug improved LFTs. This aligns with reports indicating that while drug-induced liver injury from topiramate is rare, it is essential to consider it in patients presenting with jaundice and elevated LFTs, especially when conventional causes are ruled out. Given that only a tiny percentage of patients on topiramate develop significant liver injury, this case highlights the need for vigilance in monitoring liver function in patients initiating this medication, although current literature doesn’t recommend monitoring LFTs for patients on topiramate [12].

Not to forget, as the only significant injuries that might be reported in the literature, mild impairment may have gone unnoticed. Hence, we recommend monitoring LFTs in patients who are already on other hepatotoxic medications before and after adding topiramate and vice versa, as additive or synergistic adverse effects may result in severe to fulminant liver damage.

## Conclusions

Although topiramate-induced liver injury is rare, and even rarer would be a liver injury that topiramate independently caused, the case we encountered illustrates that it is still a possibility and would have to be monitored, especially in patients with decreased hepatic reserve.
